# Supplementary material for: Simultaneous solving high-resolution structures of various enzymes from human kidney microsomes
Source: Life Sci Alliance. 2022 Nov 30;6(2):e202201580. doi: 10.26508/lsa.202201580 (PMC9713302; doi:10.26508/lsa.202201580)
Supplement: Supplementary file 1 [file LSA-2022-01580_TableS1.docx]

**Table S1. Cryo-EM data collection, processing, and refinement statistics**.

| **Data set** | **GANAB** | **ALDH1A1** | **FPA** | **BHMT** |
| --- | --- | --- | --- | --- |
| **Data collection and processing** | | | | |
| Magnification | 81,000 | | | |
| Voltage (kV) | 300 | | | |
| Electron Microscope | Krios-GIF-K3 | | | |
| Defocus range (μm) | -1.0 to -2.5 | | | |
| Total exposure time (s) | 3.5 | | | |
| Energy filter width (eV) | 20 | | | |
| Pixel size (Å) | 1.07 | | | |
| Total dose (e^-^/Å^2^) | 39 | | | |
| Number of frames | 52 | | | |
| Dose rate (e^-^/phys. Pixel/s) | 11.14 | | | |
| No. of initial micrographs | 6,679 | 9,267 | | |
| No. of initial particles | 5,568,989 | 13,854,193 | | |
| No. of final particles | 180,2286 | 95,743 | 126,024 | 471,301 |
| Symmetry | C1 | C2 | D2 | D2 |
| GSFSC Resolution (Å)  FSC threshold (0.143) | 2.88 | 2.84 | 2.80 | 2.62 |
| **Refinement** |  |  |  |  |
| Model resolution cut-off (Å) | 2.88 | 2.84 | 2.80 | 2.62 |
| Model composition |  |  |  |  |
| No. of Protein residues | 962 | 1956 | 1372 | 1358 |
| No. ligands | 6 | 0 | 0 | 0 |
| RMSD^a^ |  |  |  |  |
| Bond lengths (Å) | 0.004 | 0.004 | 0.004 | 0.004 |
| Bond angles (°) | 0.835 | 1.021 | 0.628 | 0.783 |
| **Validation** |  |  |  |  |
| MolProbity score | 2.44 | 1.90 | 1.93 | 1.64 |
| Clash score | 8.43 | 5.72 | 6.47 | 4.82 |
| Ramachandran plot (%) |  |  |  |  |
| Favored (%) | 93.5 | 96.25 | 94.28 | 94.83 |
| Allowed (%) | 6.5 | 3.75 | 5.72 | 5.17 |
| Disallowed (%) | 0 | 0 | 0 | 0 |
| CC^b^ Mask | 0.79 | 0.73 | 0.81 | 0.72 |

^a^root mean square deviation

^b^correlation coefficient
