# Supplementary material for: Simultaneous solving high-resolution structures of various enzymes from human kidney microsomes
Source: Life Sci Alliance. 2022 Nov 30;6(2):e202201580. doi: 10.26508/lsa.202201580 (PMC9713302; doi:10.26508/lsa.202201580)
Supplement: Supplementary file 2 [file LSA-2022-01580_TableS2.docx]

**Table S2. Human kidney enzymes.**

**A. Protein sample in** **250-650 kDa range.**

| **Hit** | **Score** | **Coverage** | **Mass**  **(Avg, Da)** | **Peptides Identified** | **Spectral Matches** | **Description** |
| --- | --- | --- | --- | --- | --- | --- |
| 1 | 3286 | 74% | 92753.46 | 82 | 335 | Endoplasmin |
| 2 | 2044 | 64% | 72446.52 | 54 | 151 | Endoplasmic reticulum chaperone BiP |
| **3** | **2038** | **68%** | **107329.6** | **59** | **201** | **GANAB_Neutral alpha-glucosidase AB** |
| 4 | 1643 | 65% | 96828.4 | 51 | 163 | E9PKU7_Neutral alpha-glucosidase AB |
| 5 | 1406 | 64% | 85058.5 | 49 | 110 | Heat shock protein HSP 90 |
| **6** | **1143** | **52%** | **60394.95** | **31** | **99** | **Glucosidase 2 subunit beta** |
| 7 | 1141 | 46% | 111562.8 | 41 | 74 | Hypoxia up-regulated protein 1 |
| 8 | 1139 | 49% | 193381.9 | 71 | 110 | Clathrin heavy chain 1 |
| 9 | 901 | 48% | 83605.93 | 32 | 67 | Heat shock protein HSP 90-beta |
| 10 | 889 | 55% | 83740.77 | 34 | 55 | Trifunctional enzyme subunit alpha |

**B. Protein sample in 100-200 kDa range.**

| **Hit** | **Score** | **Coverage** | **Mass**  **(Avg, Da)** | **Peptides Identified** | **Spectral Matches** | **Description** |
| --- | --- | --- | --- | --- | --- | --- |
| 1 | 2160 | 77% | 60154.294 | 55 | 197 | disulfide-isomerase |
| 2 | 1766 | 62% | 72446.515 | 53 | 120 | Endoplasmic reticulum chaperone BiP |
| **3** | **1410** | **77%** | **45454.599** | **38** | **112** | **Betaine--homocysteine S-methyltransferase 1** |
| **4** | **1393** | **68%** | **39986.236** | **27** | **134** | **Fructose-bisphosphate aldolase B** |
| 5 | 1195 | 75% | 36923.512 | 31 | 135 | L-lactate dehydrogenase B |
| 6 | 1017 | 49% | 73274.237 | 35 | 74 | Protein disulfide-isomerase A4 |
| 7 | 1001 | 74% | 37241.612 | 32 | 77 | Fructose-1 6-bisphosphatase 1 |
| 8 | 977 | 49% | 91968.713 | 37 | 60 | Endoplasmin |
| 9 | 973 | 41% | 124369.041 | 46 | 63 | Vinculin |
| 10 | 913 | 60% | 48520.333 | 24 | 69 | disulfide-isomerase A6 |
| … |  |  |  |  |  |  |
| **23** | **631** | **60%** | **55489.04** | **23** | **42** | **Aldehyde dehydrogenase 1A1** |
